# Warfarin: better the devil you know

**Published:** 2017

**Authors:** Marc Blockman

## Introduction

Long-term anticoagulation with warfarin is recommended for patients with atrial fibrillation (AF), valvular heart disease and pulmonary embolus, as these conditions significantly increase the risk for thromboembolic complications.^1^ AF for example increases the risk for ischaemic stroke four- to five-fold.^1^

Warfarin has high efficacy in the prevention and treatment of thromboembolic disease.^2^ In AF patients, for example, warfarin reduces stroke risk by 64% compared with placebo or no treatment (absolute risk reduction 2.7% for primary prevention, 8.4% for secondary prevention), and by 38% when compared to aspirin (absolute risk reduction 0.7% primary prevention, 7.0% secondary prevention).^3^

Importantly, for clinical practice, warfarin has a narrow therapeutic window and requires regular monitoring in the form of routine international normalised ratio (INR) measurements. It has an unpredictable pharmacokinetic/pharmacodynamic (PK/PD) profile, and to optimise efficacy and avoid toxicity, INR monitoring is essential.

Sub-therapeutic warfarin doses increase the risk of thrombus formation, while excess anticoagulation will increase the probability of a life-threatening bleed.^4^ Therefore, meticulous control and monitoring is required throughout treatment.

Warfarin causes significant morbidity and is among the top drugs leading to adverse drug reactions.^5^ The risk of major bleeding depends on the patient group and can range from 0.75 to 10.0% per annum.^6^-^8^

In South Africa, bleeding due to warfarin is among the top five adverse drug reactions (ADRs) resulting in hospital admission.^9^ A multicentre, hospital-based survey in South African medical wards to determine the burden of ADRs resulting in admission and death revealed that ADRs accounted for 8.4% of medical admissions and 2.9% of deaths.^10^ In this study, haemorrhage was the fourth most common cause, with warfarin accounting for 68% of these bleeds.^10^

It is difficult to predict who is at increased risk for toxicity. Many factors result in the inconsistent response to warfarin therapy. These include its narrow therapeutic window, unpredictable dose response, numerous drug–drug interactions (importantly, non-steroidal anti-inflammatory drugs, rifampicin and the enzyme-inducing anti-epileptics), diet containing high levels of vitamin K, and patient co-morbid conditions.^2^,^11^,^12^

In a South African black population, genetic modifications in cytochrome P450 2C9 and vitamin K epoxide reductase subunit 1 resulted in approximately 45% of warfarin dosage variability.^13^ Further research is required to establish whether routine genetic testing and dose adjustment will lead to improved outcomes when using warfarin.

Patient non-adherence and prescriber fear are important causes of INR variability.^4^ The Randomized Evaluation of Long-Term Anticoagulation Therapy (RE-LY) study found that adherence to a standardised warfarin dosing algorithm improved patient control.^14^

The INR is used as a surrogate for treatment success. Patients’ INRs are routinely measured and used to assess the time in the therapeutic range (TTR). TTR is defined as the duration of time in which the patient’s INR values were within a desired range. TTR strongly associates with bleeding and thromboembolic risk, namely, a high TTR correlates with reduced risk of both thromboembolic complications and major bleeding.^4^,^11^,^15^

A study in patients with AF receiving warfarin found that even a small 7% improvement in TTR reduced major haemorrhage rates by one event per 100 patient years, and a 12% increase in TTR reduced the thromboembolic rate by one event per 100 patient years.^16^ It is suggested that INR monitoring clinics aim for a TTR between 70 and 80% to optimise benefits and reduce patient harm.^16^,^17^

A post hoc analysis of the ACTIVE W study, which assessed dual antiplatelet therapy versus warfarin in patients with AF, found a mean TTR of 63.4%. Despite patients being managed in the controlled environment of this clinical trial, the South Africa cohort had a mean TTR of 46.3%; well below the widely accepted range.^17^ Countries that achieved a TTR of close to 75% had improved clinical benefits from warfarin therapy.^17^

Newer agents have been compared to warfarin in patients with AF. The Africa cohort of the RE-LY study had a TTR of 58% compared to the overall population TTR of 64%.^15^ The South African patient population of the ROCKET-AF study had a TTR of 54.8%.^18^ Once again, the outcomes of the South African cohort within the ROCKET-AF trial emphasise that despite being evaluated under clinical trial conditions, there are challenges to achieving therapeutic TTRs. Unfortunately, newer warfarin dosing strategies (computer-aided dosing, specialitypharmacy clinics and genotype-guided dosing) have shown only modest improvements in TTR.^19^

In conclusion, warfarin remains an important agent for the prevention of thrombosis and thromboembolism in highrisk patients. Despite its clinically significant effectiveness, its unpredictable bleeding risk must be respected. Before committing to its prescription, clinicians must recognise and mitigate which factors may contribute to this risk. Regular INR monitoring and patient education can dramatically reduce this risk.
